# Gold Nanoparticles Synthesized by an Aqueous Extract of *Codium tomentosum* as Potential Antitumoral Enhancers of Gemcitabine

**DOI:** 10.3390/md21010020

**Published:** 2022-12-27

**Authors:** Noelia González-Ballesteros, Immacolata Maietta, Raquel Rey-Méndez, M. Carmen Rodríguez-Argüelles, Mariano Lastra-Valdor, Antonella Cavazza, Maria Grimaldi, Franca Bigi, Rosana Simón-Vázquez

**Affiliations:** 1 CINBIO, Departamento de Química Inorgánica, Universidade de Vigo, 36310 Vigo, Spain; 2 CINBIO, Inmunology Group, Universidade de Vigo, 36310 Vigo, Spain; 3 Centro de Investigación Marina, Universidade de Vigo, 36331 Vigo, Spain; 4 Dipartimento Scienze Chimiche, della Vita e della Sostenibilità Ambientale, Università di Parma, 43124 Parma, Italy; 5 Institute of Materials for Electronics and Magnetism, National Research Council, 43124 Parma, Italy

**Keywords:** *Codium tomentosum*, gold nanoparticles, green synthesis, antitumoral, gemcitabine enhancer, HEPG-2, BxPC-3

## Abstract

Cancer still poses a global threat, since a lot of tumors remain untreatable despite all the available chemotherapeutic drugs, whose side effects, it must also be noted, still raise concerns. The antitumoral properties of marine seaweeds make them a potential source of new, less toxic, and more active antitumoral agents. Furthermore, these natural extracts can be combined with nanotechnology to increase their efficacy and improve targeting. In this work, a *Codium tomentosum* (CT) aqueous extract was employed for the green synthesis of gold nanoparticles (Au@CT). The complete characterization of Au@CT was performed by UV-Vis spectroscopy, Fourier transform infrared (FTIR) spectroscopy, Zeta potential, electron microscopy, X-ray powder diffraction (XRD), high-performance steric exclusion chromatography (HPSEC), and by the determination of their antioxidant capacity. The antiproliferative activity of Au@CT was then tested in hepatic (HEPG-2) and pancreatic (BxPC-3) cell lines. Their potential capacity as enhancers of gemcitabine, a drug frequently used to treat both types of tumors, was also tested. The activity of Au@CT was compared to the activity of the CT extract alone. A synergistic effect with gemcitabine was proven for HEPG-2. Our results showed that gold nanoparticles synthesized from seaweed extracts with antitumoral activity could be a good gemcitabine enhancer.

## 1. Introduction

Cancer is one of the main causes of death worldwide. In 2020, 19.3 million new cancer cases were diagnosed, and 10.0 million cancer deaths were estimated. Despite the relevant arsenal of chemotherapeutic drugs available at present and their success in treating and curing different types of cancer, there are still many tumors, such as pancreatic and hepatic ones, with a poor or limited response to these therapeutic agents [1]. The main reasons for this low efficacy in these tumors are both the diagnosis in advanced stages and the rapid development of drug resistance mechanisms [2,3].

Another common limitation of chemotherapy is its associated toxicity. Some drugs can induce organ damage or even trigger the metastatic process [4,5]. For this reason, the search for natural compounds that improve the therapeutic efficacy of conventional antitumoral therapies and reduce their side effects is a relevant area of research [6,7]. 

Marine seaweeds include a variety of different species, characterized by different compositions and biological properties, and they are a good natural source of potential antitumoral agents [8]. In particular, fucoidan, a sulphated polysaccharide from brown seaweeds, is the most studied seaweed compound with associated antitumoral activity [9]. Fucoidan is capable of interfering with different cellular pathways that are upregulated in tumor cells, such as PI3K/AKT or MAPK. However, sulphate polysaccharides from green and red seaweeds, such as carrageenan, and other seaweed compounds such as carotenoids, polyunsaturated fatty acids (PUFAs), or proteins have also shown potential antitumoral effects [8,10]. These compounds are able to induce cell cycle arrest or oxidative stress-mediated apoptosis in tumor cells, among other antitumoral mechanisms [8].

Seaweed compounds can also enhance the therapeutic efficacy of conventional chemotherapy drugs and reduce the associated side effects. For instance, the intraperitoneal administration (i.p.) of aqueous extracts from the brown alga *Padina pavonica* and the red alga *Jania rubens* increased the antitumoral effect of cisplatin in a mouse model of Ehrlich ascites carcinoma (EAC), inducing the activation of antitumoral immunity, while reducing systemic toxicity [11]. Similarly, oral administration of an ethanolic extract of *Sargassum fusiforme* significantly reduced the hepatotoxicity and nephrotoxicity induced by cisplatin in rats [12]. For these reasons, seaweed compounds are powerful complementary therapeutic agents for cancer treatment.

The use of nanomaterials to increase the targeting of antitumoral agents to different tumors has proven to be an efficient strategy. In fact, a relevant proportion of nanomedicines that have reached the market are antitumoral agents, and more nano-formulations are expected in the future that combine chemo- and immuno-therapeutic agents [13,14]. 

Particularly, gold nanoparticles (AuNPs) are one of the most widely studied metal nanomaterials in the field of biomedicine. They owe their popularity to their unique optical, chemical, and biological properties, which provide certain advantages over other nanoparticles [15]. In cancer therapy, AuNPs have shown both a great potential as nanocarriers for the targeted release of chemotherapeutic agents into the tumoral cells [16] and an intrinsic inhibitory activity against different tumor cell lines [17,18]. Besides, the combination of chemotherapeutic agents with AuNPs can induce a synergistic antitumoral effect. For instance, AuNPs were able to enhance the antitumoral effect of cisplatin in a mouse model of colorectal cancer by decompressing the vessels that surrounded the tumor [19]. The green synthesis of AuNPs with other natural antitumoral agents, such as algae extracts, could not only enhance the therapeutic effect of the nanomaterials obtained, but also increase their biocompatibility due to the fact that this green method of synthesis implies lower levels of toxicity than traditional methods [20]. 

In this work, which continues our research line on the green synthesis of metal nanoparticles led by natural extracts [21,22], an aqueous extract of the green seaweed *Codium tomentosum* Stackhouse (CT) was used to synthesize gold nanoparticles (Au@CT). CT is a green macroalgae native to the North-East Atlantic Ocean, found on exposed shores in deep rock pools, and in the lower intertidal and subtidal zone. CT is a rich source of polysaccharides, fatty acids, and other bioactive compounds, such as antioxidants, which makes this species a valuable resource with nutritional and health benefits [23,24]. CT can be easily obtained by aquaculture [25], which could be an advantage for the production of bioactive extracts in a controlled environment. CT possesses relevant neuroprotective activity [26,27,28,29], but its antitumoral activity has not been thoroughly characterized. To the best of our knowledge, CT extracts have only been tested in Caco-2 cells (human colon adenocarcinoma), showing an IC_50_ of 200 µg/mL in this cell line [30]. However, the potential antitumoral effect of this seaweed species has not been tested in other types of tumors. Interestingly, the biocompatibility of polysaccharides extracted from CT has been tested, showing no toxicity against mouse fibroblast cells (L929) [31]. 

The potential antiproliferative effect of Au@CT was tested in a hepatic (HEPG-2) and a pancreatic (BxPC-3) cell line, alone or in combination with gemcitabine, a nucleoside analogue commonly used to treat hepatic and pancreatic cancer [32,33]. We also tested the therapeutic efficacy of the CT extract alone. 

## 2. Results and Discussion 

### 2.1. Synthesis and Spectroscopic Characterization of Au@CT

An aqueous extract of CT was employed for the green synthesis of AuNPs. To optimize the reaction conditions, several experiments were performed, varying some parameters, in particular, concentrations of the extract and metal salt, the temperature, and the reaction time. In all cases, the reactions were monitored by color change, from pale-yellow to purple, and by UV-Vis spectroscopy. The final conditions chosen as optimal were a concentration of the extract of 1 g/mL and a concentration of gold of 0.4 mM at room temperature for 24 h. These conditions were employed for the full characterization of Au@CT and the evaluation of the antiproliferative activity.

**Figure 1:**
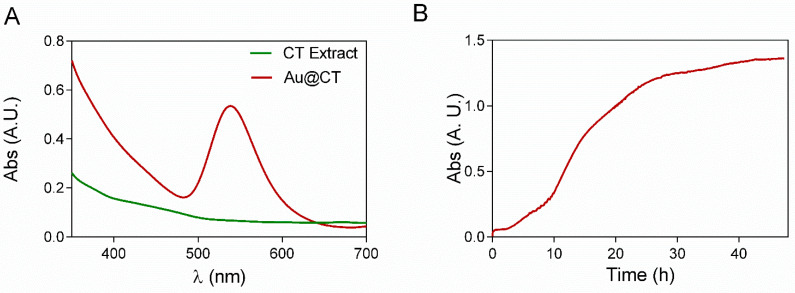
(**A**) UV-Vis spectra of CT extract and Au@CT and (**B**) time course absorbance measurements of Au@CT.

The UV-Vis spectrum of Au@CT shows the presence of the SPR band of AuNPs with λ_max_ at 539 nm, while the spectrum of the CT extract shows no absorption band in that region (Figure 1A). UV-Vis absorption at the wavelength of the SRP band was recorded every 10 s, as shown in Figure 1B, to analyze in detail the process of formation of AuNPs. The reaction went through three different identifiable stages. The activation process took place between 0 and 2.5 h, when there was no appreciable change in the absorbance of the sample. The increase of absorbance started at 2.5 h and continued until 24 h. This stage would correspond to the nucleation and growth steps, and it is coincident with the observable change in color. After 24 h, the increase in absorbance ceased, confirming the end of the reaction. The measurements were stopped after 50 h. 

The stability and the surface charge of the nanoparticles obtained were analyzed by the measurement of Zeta potential. A value of −22.06 ± 0.58 mV was obtained, indicating that Au@CT possess a negative surface charge (Figure S1A, Supplementary Material). According to other reports, this value suggests good stability for the colloidal suspension. Au@CT samples were proven to be stable for more than three months when preserved at 4 °C, as confirmed by UV-Vis spectra measurements (Figure S1B). 

**Figure 2:**
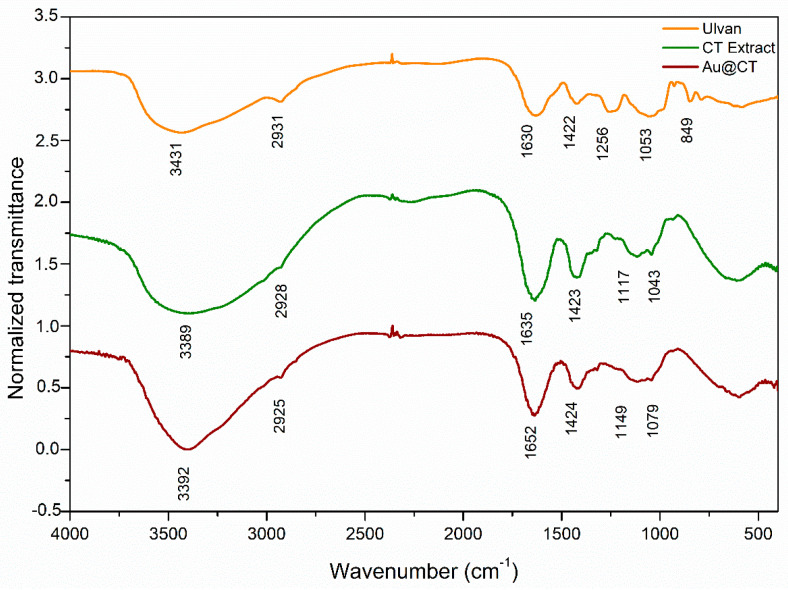
Fourier transform infrared (FTIR) spectra of commercial ulvan (orange line), CT extract (green line), and Au@CT (red line).

Infrared spectra of the CT extract before and after the synthesis of Au@CT were obtained and compared with the spectrum of ulvan, a commercially available polysaccharide extracted from green seaweeds. Bands were assigned following other studies on the composition of different *Codium* spp. [12,34,35,36,37]. All the spectra are shown in Figure 2. 

The spectrum of ulvan reveals the typical profile of other polysaccharides: (a) At 3431 cm^−1^, the spectrum shows the strong broad band of NH stretching vibrations and OH stretching vibrations of the hydroxyl group of carbohydrates, associated with intermolecular and intramolecular hydrogen bonding [34]. (b) At 2931 cm^−1^, the week band of aliphatic C-H groups’ stretching vibrations can be seen. (c) The two bands at 1630 and 1422 cm^−1^ are associated, respectively, with asymmetrical and symmetrical stretching of the carboxylate groups [37]. (d) The bands in the region between 1200 and 970 cm^−1^ are assigned to C-C and C-O stretching and C-O-C glycosidic vibrations, which are common to natural polysaccharides [12,38,39]. (e) Finally, the presence of sulphate groups in the sample was confirmed by the band at 840 cm^−1^, attributed to C-O-S bending vibration, while the band at 1250 cm^−1^ can be assigned to S-O stretching vibration of sulphate esters [40]. 

Comparison between the spectra of both the CT extract and ulvan revealed some notable differences. A shift towards lower wavelengths can be seen in the band at 3431 cm^−1^, while the band at 1630 cm^−1^ shifts toward higher wavelengths. These variations can be explained since ulvan is found in the cell wall of green algae, closely associated with proteins and other biomolecules [41]. Therefore, the CT extract is expected to have these different molecules in association with ulvan. Another significant difference between the spectrum of the CT extract and that of ulvan can be seen in the band at 1200 cm^−1^, associated with ester sulphate. In the case of the CT extract, this band almost disappeared, which would be in line with the proposal of Fernández et al., who showed how the FTIR spectra of the polysaccharides extracted from the cell wall of different *Codium* spp. vary depending on the degree of sulphurization of the polysaccharides [37]. Finally, the comparison between the spectra of the CT extract before and after the synthesis of Au@CT showed minor shifts in the position, shape, and intensity of the bands at 3393 cm^−1^, as well as in the region between 1100 and 1000 cm^−1^, which suggests the involvement of the functional groups assigned to these bands in the reducing and stabilizing processes that take place during the synthesis. 

### 2.2. Carbohydrate Analysis

As is known, the sugar fraction is the main component of seaweed extracts and contains both soluble and insoluble fibers. Its composition, in terms of molecular weight distribution and type of carbohydrates occurring, is quite different when considering different seaweed species. However, a high variability between samples grown under different environmental conditions has been reported [42]. In previous works, a punctual analysis of seaweed extracts was performed by size exclusion liquid chromatography, allowing the detection of the entire sugar pattern. It could be seen that the chromatographic profiles recorded for different species showed high variability. *Ulva intestinalis* showed the preponderance of sugars having a molecular weight higher than 150 kDa [43], whereas in *Sargassum muticum,* shorter chains were detected, with majority of species having a molecular mass lower than 150 kDa [44]. 

**Figure 3:**
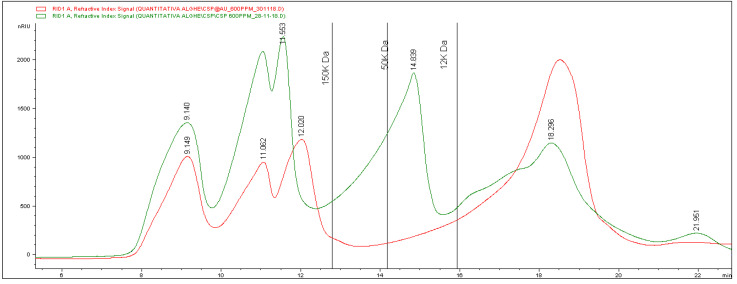
Comparison of the carbohydrate profile obtained from the analysis of CT extract (green line), and of the same extract after Au@CT formation (red line).

In Figure 3, the profile of the CT extract is shown in green. It reveals the presence of a complex mixture of molecules with different chain lengths, distributed at different retention times. In detail, at least six bands eluting at different times can be detected. The vertical lines in the graph indicate the retention times of calibrating standards injected with the aim of attributing a molecular weight to the bands. Based on standard elution times, it is possible to attest the presence of three different groups of analytes having a molecular mass higher than 150 kDa. A large band with molecules from more than 50 to 12 kDa can be seen, and a last broad signal was finally eluted and corresponds to smaller compounds.

From the information obtained, it is possible to calculate an average molecular weight of about 83 kDa.

Quantitative data for each separate fraction were obtained by means of a calibration curve built using standard solutions at different concentrations. The results are reported in Table 1, showing the amount of each class of molecular weight expressed as mg of compound per g of lyophilized extract.

As for the involvement of the different fractions in the formation of the nanoparticles, the red line in Figure 3 corresponds to the chromatogram obtained from the analysis of the extract after the reaction. The comparison between the two recorded traces reveals that significant changes occurred after the reaction. In particular, there was a massive reduction (about 85%) of the fraction related to high molecular masses. Besides, the band between 50 and 12 kDa completely disappeared, whereas the last one showed a significant increase (about 54%). The calculation of the average mass molecular weight after the formation of the nanoparticles gave a result of about 70 kDa, which is significantly lower than the value found in the original extract (83 kDa). These data allow us to hypothesize the involvement of longer chains in the process of formation and stabilization of the nanoparticles.

### 2.3. Transmission Electron Microscopy (TEM) Characterization of Au@CT

**Figure 4:**
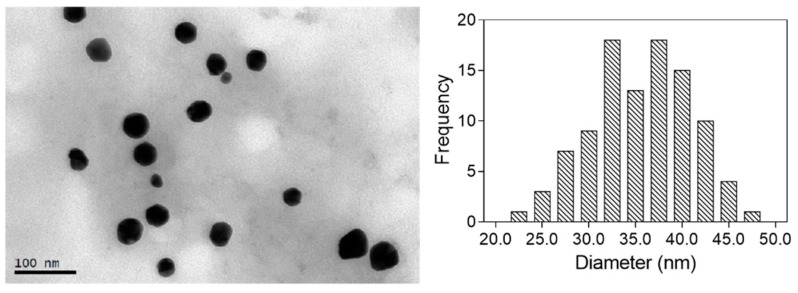
TEM image of Au@CT with its corresponding size distribution histogram.

TEM was used for the characterization of the size and shape of the nanoparticles obtained. The corresponding size distribution histogram was also calculated by measuring a significant number of nanoparticles (>100). The images obtained for the optimal nanoparticles are shown in Figure 4. All the particles analyzed were spherical, with a mean diameter of 34.5 ± 5.6 nm. 

**Figure 5:**
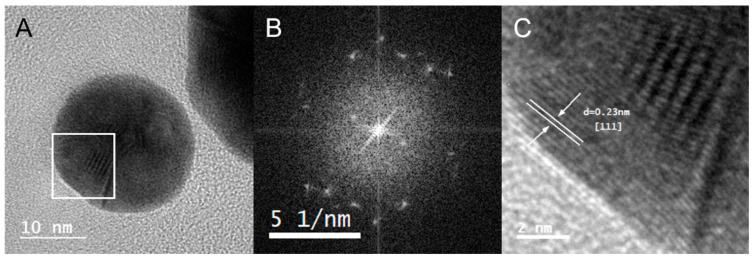
(**A**) HRTEM images of Au@CT with (**B**) its corresponding Fourier transform and (**C**) the amplification of the selected area, showing the interplanar distance of the crystalline structure with the calculated d-spacing and their corresponding Miller index.

The crystalline nature of Au@CT was analyzed by high-resolution transmission electron microscopy (HRTEM). The analysis of Fourier transform (Figure 5) confirmed the polycrystalline structure of the nanoparticles investigated, since they showed several disorder points instead of an ordered pattern and they displayed several crystal domains. HRTEM images of Au@CT and their corresponding Fourier transform can be observed in Figure 5. In the amplified region shown in the image, the d-spacing was calculated and the associated Miller index was obtained by comparison with tabulated data. The results showed a preferential d-spacing of 0.23 nm, which corresponds to a Miller index (111), compatible with the face-centered cubic structure of gold.

**Figure 6:**
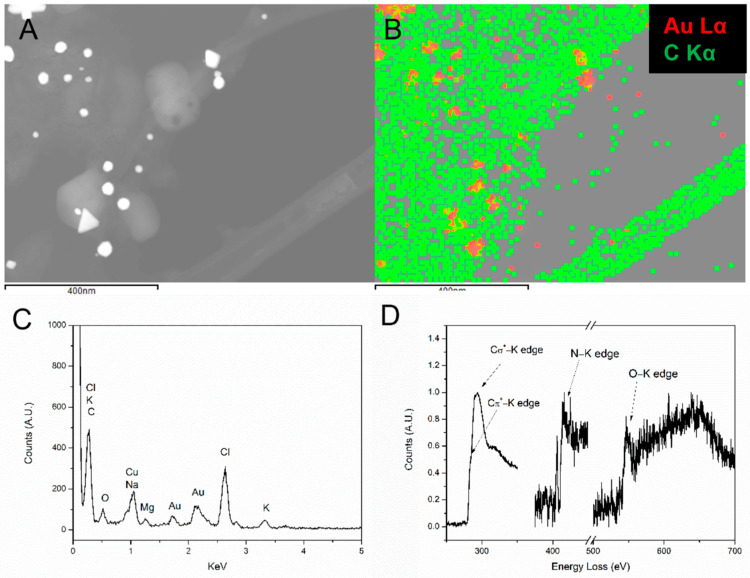
(**A**) DF-STEM image, (**B**) mapping, (**C**) EDX spectrum, and (**D**) EELS spectrum of Au@CT.

A dark-field scanning transmission electron microscopy (DF-STEM) image was acquired, as shown in Figure 6A. In this image, AuNPs appear with bright contrast, surrounded by a dark mass. As confirmed by mapping (Figure 6B), energy-dispersive X-ray analysis (EDX) (Figure 6C), and electron energy loss spectroscopy (Figure 6D), this mass corresponds to the organic matter of the CT extract.

The EDX spectrum (Figure 6C) shows the elemental composition of the sample. Besides gold, elements naturally occurring in seaweed, such as carbon, chlorine, magnesium, oxygen, potassium, and sodium, can be found. It has been reported that these elements are present in the composition of CT [34]. The signal of copper could be due to the copper grid where the sample was prepared. However, in the case of Au@CT, it has been reported that CT possesses high levels of copper, so it cannot be excluded from the composition of the extract [45,46].

Mapping analysis was performed selecting carbon and gold. It can be noted that gold is accumulated in the nanoparticles, and does not appear in the extract, which suggests the complete reduction of the gold salt into AuNPs. This was further confirmed by ICP-OES. 

Finally, EELS is more appropriate than EDX for the analysis of elements with low atomic numbers. Therefore, to confirm the organic nature of the mass observed, EELS was performed using a holey carbon grid and analyzing areas of the sample over a hole to eliminate the contributions of the grid. By doing this, the characteristic edges of carbon (284 KeV), nitrogen (401 KeV), and oxygen (532 KeV) were obtained (Figure 6D). 

### 2.4. X-ray Powder Diffraction (XRD)

**Figure 7:**
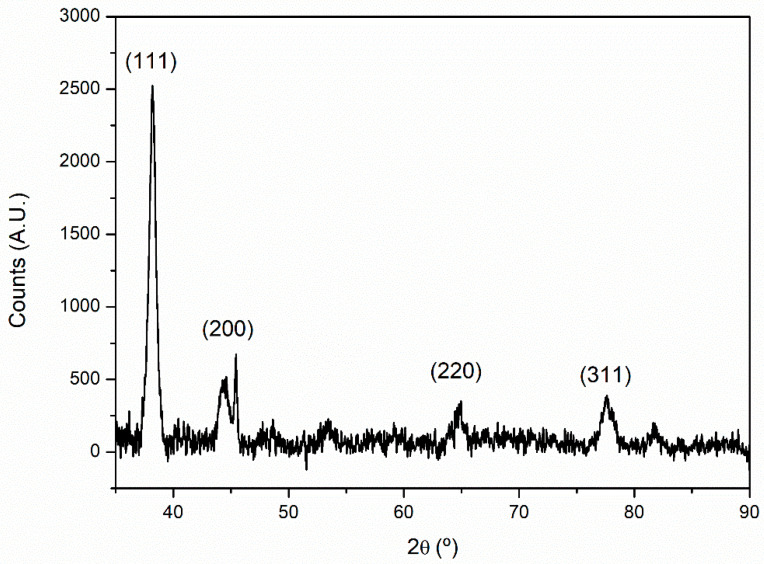
X-ray powder diffractogram for Au@CT.

The crystallinity of the sample was further analyzed by XRD. The diffractogram obtained (Figure 7) shows that the samples analyzed were crystalline, displaying the characteristic peaks that correspond to the face-centered cubic structure of gold (JCPDS file No. 00-004-0784). The following peaks were identified, with position 2θ = 38.1°, 44.4°, 64.9°, and 77.5°, corresponding to a planar interspacing of 0.235, 0.204. 0.144, and 0.123 nm, respectively, and can be assigned to the planes (111), (200), (220), and (311) of the FCC gold structure [22,47]. 

### 2.5. In Vitro Antioxidant Activity

**Figure 8:**
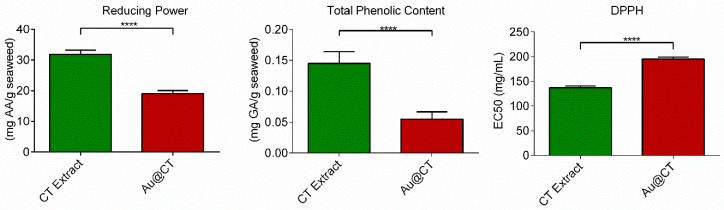
Reducing power, total phenolic content, and DPPH scavenging activity of CT extract (green) and Au@CT (red) (**** *p* ≤ 0.0001).

To assess the potential of the CT extract to act as the reducing agent in the synthesis of AuNPs, its in vitro antioxidant activity was studied by means of three different assays: reducing activity, total phenolic content (TPC), and 2,2-diphenyl-1-picrylhydrazyl (DPPH) scavenging activity. Figure 8 summarizes the results obtained.

The results confirmed the presence of reductants on the CT extract and its capability of reducing the metal salt to nanoparticles. A comparison with data from the literature poses certain problems due to the current lack of a standardized method for the determination of the reducing power. However, to the best of our knowledge, there are no previous studies on the reducing capacity of CT extracts. Comparison between this extract and extracts obtained from other algae showed that the CT extract possesses similar reducing capacity to that of the extracts prepared with the green seaweeds *Ulva lactuca* and *Ulva intestinalis* [43,48]. 

It has been shown that there is a direct correlation between TPC and antioxidant activity. Matching the results of the reducing capacity assay, the CT extract also presents high TPC. In this case, there are previous reports on the TPC of CT extracts. However, data comparison is challenging due to the lack of standardized methods and because the results are greatly affected by extraction and assay conditions [30,34,46,49,50].

Finally, DPPH is a stable free radical at room temperature that accepts an electron or hydrogen radical to become a stable diamagnetic molecule [51]. The method used has been extensively applied for the screening of antioxidants present in natural extracts. The results obtained revealed that the CT extract contained free radical scavengers, which reacted with DPPH radicals by their electron-donating ability, with an EC_50_ value of 136.9 ± 3.6 mg/mL. When this result was compared with results obtained by other authors, the values obtained in the present study were quite different. This could be explained by the fact that, as several studies have argued, both the use of different solvents in the extraction and the method employed for the determination of scavenging activity interfere with the DPPH radical [52]. Variation in solvent polarity could modify the efficiency to retrieve a specific group of compounds and, consequently, influence the antioxidant properties of the extracts [53]. 

The reducing power, total phenolic content, and DPPH scavenging activity of the extract were also determined after the synthesis of Au@CT (Figure 8). In the case of the reducing power and total phenolic content, a significant decrease in the values obtained can be appreciated. Au@CT possess almost half the reducing power of CT and 3 times less than TPC. This seems to indicate that the phenolic compounds present in the CT extract actively contribute to the reduction process that takes place during the synthesis of the nanoparticles. In the case of the DPPH, the increase observed in the EC_50_ value also indicates a decrease in the radical scavenging activity of the extract after the synthesis of Au@CT. Similar results were appreciated in a previous study on the synthesis of AuNPs led by red algae species, where a significant decrease in the reducing power, TPC, and DPPH scavenging activity was noted in the particles synthesized by *Chondrus crispus* and *Gelidium corneum* [21].

### 2.6. Antiproliferative Effect of Au@CT and CT Extract on Human Hepatic and Pancreatic Cell Lines

To evaluate the antiproliferative effect of Au@CT and the CT extract, a kinetic cell viability assay was conducted. A pancreatic cell line (BxPC-3) and a hepatic cell line (HEPG-2) were used, being the cells incubated with different concentrations of Au@CT (0.4, 4, 40 M) and CT extract at equivalent concentrations (1, 10, 100 mg/mL) for more than 96 h. 

**Figure 9:**
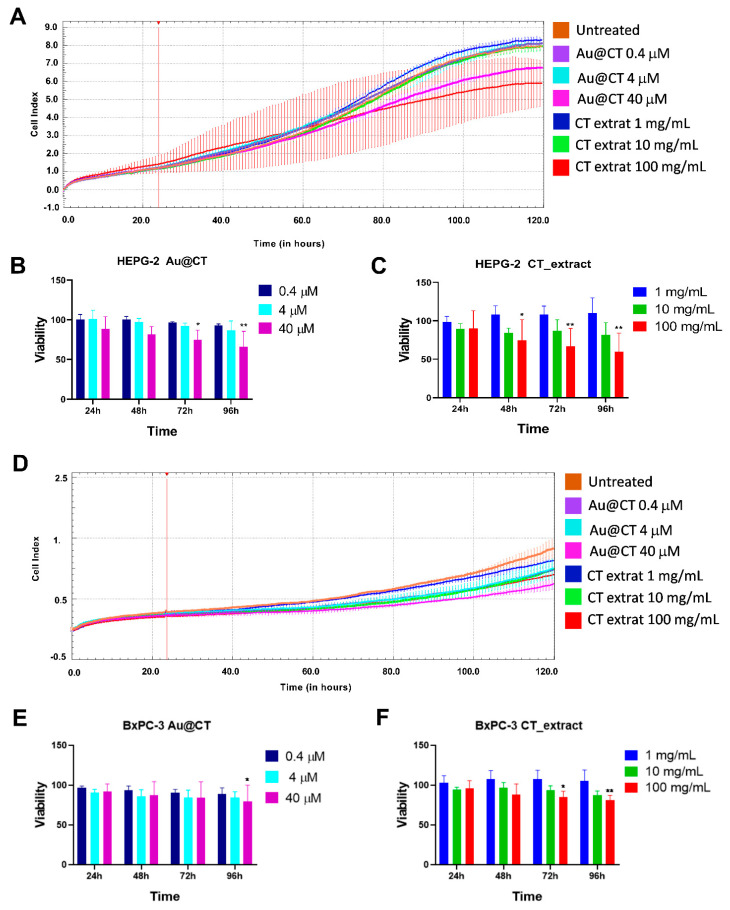
(**A**,**D**) Kinetics of the viability of BxPC-3 and HEPG-2 cells incubated with Au@CT and CT extract, respectively. The treatments were added after cell stabilization (vertical red lines). (**B**,**C**,**E**,**F**) Percentage of cell viability after 24, 48, 72, and 96 h of incubation (* *p* ≤ 0.05, ** *p* ≤ 0.01).

Figure 9A–C show the viability of HEPG-2 cells in real-time and at different time points. In the presence of the highest dose of Au@CT (40 µM), there was a significant decrease in cell proliferation at all the time points studied (24 up to 96 h). The antiproliferative effect of Au@CT at 40 µM was similar to the effect observed for the CT extract equivalent concentration (100 mg/mL). However, the viability was never under 60% at the concentrations tested. Similarly, the highest antiproliferative effect in the BxPC-3 cells was obtained with Au@CT and the CT extract at the highest dose tested (Figure 9D–F). The reduction in cell viability was lower than in the case of HEPG-2 cells and only significant after 96 h of incubation with Au@CT and 72 h with the CT extract. 

In summary, the HEPG-2 cell line was more sensitive to Au@CT and the CT extract than the BxPC-3 cell line. The results obtained with Au@CT were similar to those observed previously with AuNPs synthesized by using fucoidan from the brown seaweed *Padina tetrastromatica*, also in HEPG-2 cells, at a concentration of 50 µg/mL (fucoidan and gold proportion not specified) [54]. Moreover, a similar antiproliferative effect, mediated by tumor cell apoptosis, was observed for AuNPs synthesized by aqueous extracts obtained from the red seaweeds *Chondrus crispus*, *Gelidium corneum*, and *Porphyra linearis* together with carrageenan extracted from *Mastocarpus stellatus* in a monocytic cell line. However, no effect was observed in a lung epithelial cell line (A549) using equivalent concentrations to the ones used for Au@CT [21,55].

### 2.7. Synergistic Antitumoral Effect of Au@CT and CT Extract in Combination with Gemcitabine 

After the characterization of the antiproliferative effect, it was tested whether Au@CT could enhance the antitumoral effect of gemcitabine in HEPG-3 and BxPC-3 cells. The CT extract in combination with gemcitabine was also tested as a control.

Gemcitabine is a nucleoside analogue that blocks DNA replication, leading to cell cycle arrest and apoptosis [56]. Although gemcitabine is commonly used to treat hepatic and pancreatic cancer, its therapeutic effect is limited [32,33]. For this reason, finding a compound that enhances the antitumoral effect of gemcitabine could be useful to improve the therapeutic outcome of the patients.

**Figure 10:**
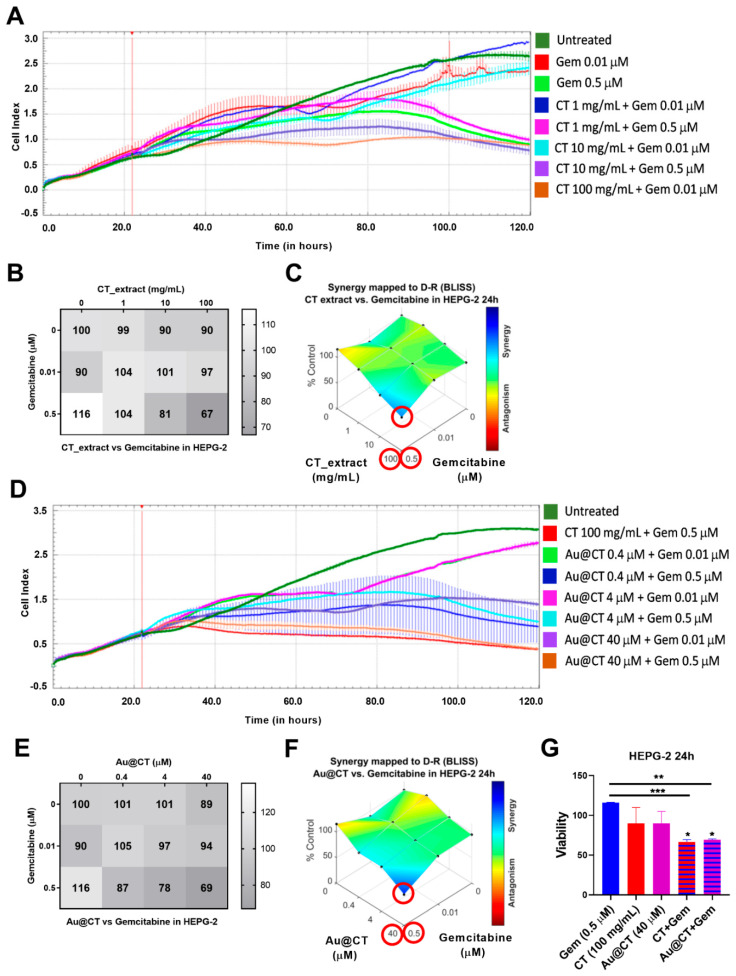
(**A**,**D**) Kinetics of cell viability of HEPG-2 incubated with Au@CT (0.4, 4, 40 µM) and CT extract (1, 10, 100 mg/mL) in combination with gemcitabine (0.01, 0.5 µM). The treatments were added after cell stabilization (vertical red lines). (**B**,**E**) Cell viability matrix. (**C**–**F**) Bliss synergy graphs. Red circles: highest synergy score and associated concentrations. (**G**) Cell viability of HEPG-2 cells with the individual drugs and the combinations at the concentrations that showed the highest synergistic effect. Statistically significant differences compared to the untreated cells (* *p* < 0.05) and compared to gemcitabine alone (** *p* < 0.01, *** *p* < 0.001).

HEPG-2 cells were incubated with different concentrations of gemcitabine (0.01 and 0.5 M), CT extract (1, 10, 100 mg/mL), and Au@CT (0.4, 4, 40 µM), alone and in combination. Cell viability was recorded with xCELLigence for 96 h (Figure 10A,D). The cell viability matrix was calculated for the different concentrations (Figure 10B,E) and the synergistic score analysis was performed by using Combenefit software (Figure 10C,F) to assess whether the combination between the two compounds at different concentrations induced a synergistic, additive, or antagonistic effect.

The results showed a synergistic effect and a reduction in cell viability of about 60% with the combination of Au@CT at 40 µM and gemcitabine at 0.5 µM after 24 h of incubation (Figure 10F,G). The extract alone at an equivalent concentration (100 mg/mL) showed a similar result (Figure 10C,G). It is remarkable that Au@CT and CT were able to induce a significant reduction of the cell viability by using a low concentration of the chemotherapeutic drug. This could be relevant to reduce the gemcitabine-associated toxicity in vivo. In addition, the antiproliferative effect was accelerated in both combinations compared to the individual compounds (gemcitabine, Au@CT, or CT extract). It must be noted that gemcitabine alone induced cell proliferation up to 24 h, and after 48 h of incubation it decreased the cell viability, which is in line with previous studies [57]. The combination with Au@CT or the CT extract was able to boost the antiproliferative effect of gemcitabine.

Consistent with our results, fucoesterol, a sterol extracted from seaweeds, enhanced the antiproliferative effect induced by 5-fluoroacil in two different colon cell lines (HT29 and HCT116), although the antiproliferative effect induced by fucoesterol alone was low (HT29) or not significant (HCT116) [58].

The enhanced antitumoral effect of Au@CT and CT extract in combination with gemcitabine could be mediated by several components of the CT extract. One of the main components of the seaweed with antitumoral activity are polysaccharides. They can block the cell cycle progression of the tumoral cells, induce apoptosis, inhibit metastasis, or trigger the activation of the immune system, among other antitumoral mechanisms [9,59]. Similarly, other components of the seaweed extracts, such as pigments or polyunsaturated fatty acids, have shown relevant antitumoral activity by interference with several signaling pathways related to cell proliferation and apoptosis. They can also enhance the apoptotic mechanisms induced by different chemotherapeutic drugs [60].

Seaweed compounds can also protect from chemotherapy-associated toxicity. For instance, a protective effect against tumor and chemotherapy-induced caquexia in bladder cancer was described for low molecular weight fucoidan (LMWF) [61]. A LMWF-supplemented diet ameliorated weight loss and muscle atrophy induced by gemcitabine and cisplatin in a mouse model of bladder cancer. 

Au@CT and the CT extract did not induce a synergistic antiproliferative effect in BxPC-3 cells (Supplementary Figure S2). This is probably due to the high therapeutic effect of gemcitabine alone in this cell line. Gemcitabine induced a strong cytotoxic effect in BxPC-3 cells, and the combination with Au@CT showed no significant differences at any of the concentrations tested. To completely discard the potential synergistic effect in pancreatic cancer, a partial gemcitabine-resistant cell line could be evaluated.

In summary, the combination of Au@CT with gemcitabine was able to enhance and to accelerate the antitumoral effect of the chemotherapeutic drug in the hepatic cell line. The effect of Au@CT was similar to the one induced by the CT extract in vitro. However, Au@CT could have an advantage in vivo over the CT extract when it comes to reaching the tumor microenvironment. 

Our study highlights the therapeutic potential in vitro of AuNPs synthesized from a bioactive seaweed extract as chemotherapeutic enhancers. Future studies to characterize the targeting and therapeutic efficacy in vivo would be an asset for the clinical translation of gold nanoparticles synthesized from bioactive extracts. In addition, the binding or encapsulation of the chemotherapeutic drug into the nanoparticles could be an efficient strategy to increase the synergistic effect while reducing the side effects.

## 3. Materials and Methods

All reagents were high quality and utilized without further purification in the assays. Milli-Q water was used at all the steps throughout the experiments, except for in vitro assays, where the extract and the nanoparticles were prepared in endotoxin-free water and sterilized using a filter of 0.22 μm to remove possible microorganisms.

### 3.1. Preparation of Aqueous CT Extract

CT samples were collected in the NW coast of Spain (42°12′2.9″ N; 8°47′6.2″ W) at the lower intertidal rocky shore. The biomass was either immediately processed or stored at −20 °C until further use. 

For the preparation of the extracts, the seaweed was thoroughly rinsed with MilliQ water to clean any residue of sea water, sand, or biota, and then left on blotting paper to remove the excess of water. The dried seaweed was weighed and cut into small fragments. The fragments were placed into a balloon connected to a refrigerant, and then boiling ultrapure water in a 1 g/mL proportion was added. The mixture was boiled at reflux for 15 min. After the mixture cooled down to room temperature, the extract was filtered using a strainer and centrifuged at 4500 rpm for 20 min. Finally, the supernatant was collected and filtered once again using a FILTER-LAB® polyethersulphone (PES) syringe filter with a pore size of 0.22 µm The filtered extract was either preserved at 4 °C for its immediate use or stored at −20 °C for later processing. 

### 3.2. Preparation of Au@CT 

The optimal conditions for the synthesis of Au@CT were found after several trials, where the concentration of extract, gold salt (Gold(III) chloride trihydrate (HAuCl_4_·3H_2_O) 99.9% Sigma-Aldrich), temperature, and reaction time were modified. The tests were, in all cases, monitored by color change and UV-Vis spectroscopy. The best conditions to obtain the narrowest particle size distribution were achieved as follows. A concentration of CT extract of 1 g/mL was kept stirring at room temperature (RT) and, to this solution, an aqueous HAuCl_4_ solution was added to reach a final gold concentration of 0.4 mM. The mixture was left to react with constant stirring at RT for 24 h. The end of the reaction was set when no further changes in the intensity of the plasmon resonance peak for AuNPs were appreciable. Measurements of pH were also performed before and after the synthesis. A significant drop in the pH of CT from pH = 7.08 before the reaction to pH = 4.12 in the Au@CT solution was observed. 

### 3.3. CT and Au@CT Characterization 

Both CT extract and Au@CT solutions were characterized by UV-Vis spectroscopy recorded in a Jasco Spectrometer V-670 in a range between 300 and 700 nm at RT. 

ZetasizerNano S (Malvern Instruments, Malvern, UK) was used for the determination of Zeta potential of Au@CT. The measurements were performed 5 times. 

FTIR spectra of CT extract before and after the synthesis of Au@CT were recorded between 4000 and 400 cm^−1^ at RT and a resolution of 4 cm^−1^ in a Jasco FT/IR-6100 spectrophotometer. Previously, samples had been prepared by placing them in an oven at 80 °C until they were dry. Then, a fine powder was obtained by grinding the samples, which was employed for the preparation of KBr pellets. 

Size exclusion liquid chromatography was chosen as the separative system to evaluate and detect the polysaccharides present in the seaweed extracts. An Agilent 1200 Series system, equipped with a refractive index detector, was used (Agilent 1260 Infinity, from Agilent Technologies Palo Alto, CA, USA). The selected column was a PL aquagel-OH 40 (300 × 7.5 mm, particle size 8 µm), and the eluting conditions were the ones previously described [43].

Solutions to be injected were obtained from dried extracts, adding water to reach a final concentration of 600 ppm. Then, the liquid was centrifuged and purified using a solid-phase extraction system (Dionex OnGuard II P cartridge) able to remove polyphenols. A volume of 100 microliters was injected 3 times.

Molecules of dextrans (Sigma Aldrich) with a molecular weight of 150, 50, and 12 kDa were chosen to obtain standard solutions to build a calibration curve to calculate molecular weight distribution. Retention time values expressed as decimal logarithm were plotted against molecular weight values to obtain a linear equation.

To perform a quantitative analysis, a calibration curve was built by using 50 kDa dextran analyzed at 5 different concentrations. This was performed three times. The corresponding linear equation was used to evaluate the amount of compound belonging to the different classes of molecular weights.

Low-magnification TEM images were acquired in a JEOL JEM1010 operated at 100 kV, while a JEOL JEM2010F field emission gun TEM, operated at 200 kV, was employed for the acquisition of HRTEM and STEM images. Data acquisition and analyses were performed using Digital Micrograph software by Gatan (Version 3.7, Gatan, Inc., Pleasanton, CA, USA). Image J software (version 1.45s, National Institutes of Health, Bethesda, MD, USA) was used for the measurement of particle diameters to build the size distribution histogram. The EDX spectrum and mapping were obtained using the coupling between the STEM unit and the EDS detector (Oxford Inca Energy 200), while EELS measurements were performed in STEM mode using a Gatan Quantum EELS GIF. For the preparation of the sample, to attain high-quality images, Au@CT were centrifuged at 10,000 rpm for 20 min to eliminate part of the organic matter of the extract. Then, a drop of Au@CT dispersion was placed directly onto a holey carbon film resting on a copper grid. 

A Perkin Elmer Optima-4300 DV ICP-OES (Perkin Elmer, Waltham, MA, USA) was used for the determination of the gold concentration using Indium as an internal standard.

The crystallinity of the Au@CT sample was analyzed by XRD using a XpertPro X-ray diffractometer (Malver Panalytical, Malvern, UK)) equipped with Cu K_α_ radiation (λ = 0.154 nm), operated at a current of 40 mV and a voltage of 40 kV. The diffractogram was acquired between the 2θ ranges of 20° to 90° and a step size of 0.013°. The samples were prepared by dropping the nanoparticles’ dispersion on a glass substrate and drying in an oven at 100 °C. 

### 3.4. In Vitro Antioxidant Activity

The antioxidant and free radical scavenging activity of the extract prior to and after the synthesis of nanoparticles were determined by three assays: reducing power, TPC, and DPPH scavenging activity. The analyses were performed following the protocols previously reported [62,63].

#### 3.4.1. Reducing Power

Here, 1.0 mL of sample, 2.5 mL of phosphate buffer (0.2 M, pH 6.6), and 2.5 mL of potassium ferricyanide (1%) were mixed and incubated at 50 °C for 20 min. Then, 2.5 mL of trichloroacetic acid (10%) was added and the mixture was centrifuged at 4000 rpm for 10 min. Next, 2.5 mL of the upper layer was mixed with 2.5 mL of water and 0.5 mL of iron(III) chloride (0.1%). The absorbance was measured at 700 nm. Ascorbic acid was employed to build a standard curve between 50 and 400 mg/L. Results are expressed as ascorbic acid equivalents (AAE) per gram of seaweed. All assays were performed in triplicate. 

#### 3.4.2. Total Phenolic Content

Here, 100 µL of sample was mixed with 2 mL of sodium carbonate (5%) and left to stand for 2 min at room temperature. Then, 100 µL of Folin–Ciocalteau reagent 50% was mixed and allowed to stand for 30 min at room temperature in the dark. The absorbance was measured at 725 nm. Gallic acid was used to build a calibration curve between 0.05 and 1 mg/mL. Results are expressed as gallic acid equivalents (GAE) per gram of seaweed. All measurements were performed in triplicate and results are expressed as mean ± standard deviation.

#### 3.4.3. DPPH Scavenging Activity

Here, 3 mL of the sample (diluted at a ratio of 1:12) was mixed with 1 mL of DPPH (0.1 mM in methanol) and allowed to stand at room temperature for 30 min. For the blank, 3 mL of MilliQ water was used instead of the sample and a sample control was also prepared by mixing 3 mL of sample with 1 mL of methanol instead of DPPH solution. The absorbance was measured at 517 nm. The capability of scavenging the DPPH radical was calculated by using the formula:

DPPH scavenging effect (% inhibition) = (1 − (A_s_ − A_s0_)/A_b_) × 100 (1)

where A_b_ is the absorbance of the blank, A_s_ is the absorbance of the samples, and A_s0_ is the absorbance of the sample control. All the tests were performed in triplicate and the results were averaged. The results are expressed as the concentration required to inhibit the radical concentration of DPPH by half (EC_50_).

### 3.5. Cell Culture

Human pancreatic (BxPC-3) and hepatic (HEPG-2) cell lines, provided by the American Type Culture Collection (ATCC), were cultured in RPMI 1640 medium and Dulbecco’s Modified Eagle Medium (DMEM), respectively (Corning, New York, NY, USA), at 37 °C and 5% CO_2_. Both culture media were supplemented with 10% fetal bovine serum (FBS, Sigma-Aldrich, St. Louis, MO, USA), 100 U/mL Penicillin, and 100 μg/mL Streptomycin (Gibco, New York, NY, USA). Cells were sub-cultured every 2–3 days.

### 3.6. Cell Viability Determined by xCELLigence 

BxPC-3 and HEPG-2 cells were seeded in 16-well E-Plates (ACEA, Biosciences, San Diego, CA, USA) at a density of 6 × 10^3^ cells/well and 1.5 × 10^4^ cells/well, respectively, in a volume of 100 µL. After a 24 h resting period to allow the adherence of the cells to the plates, Au@CT at 0.4, 4, and 40 µM and CT extract at 1, 10, and 100 mg/mL were added to different wells by adding 100 µL/well of Au@CT or CT at 2-fold the final concentration. Cell viability and proliferation were dynamically monitored with an xCELLigence real-time cell analyzer (RTCA, Agilent, Santa Clara, CA, USA) system for 96 h. The equipment measures impedance to monitor cell growth and cell death in real time. The electrical signal, which is proportional to the number of cells, is transformed into numerical values, represented as a Cell Index. Au@CT and the CT extract alone in culture media were also monitored to discard any interference. For the characterization of the potential synergistic effect in both cell lines, Au@CT and the CT extract at equivalent concentrations were tested in combination with gemcitabine at 0.01 and 0.5 µM as a physical mixture following the procedure described above; namely, by adding 50 µL/well of a stock prepared at 4-fold the final concentration of each component (CT extract or Au@CT and gemcitabine).

### 3.7. Analysis of the Synergistic Antiproliferative Effect 

The synergistic therapeutic effect between Au@CT and gemcitabine, on the one hand, and the CT extract and gemcitabine, on the other, was calculated by using Combenefit software and the Bliss Independence model. According to the software developers, a score > 25 means a synergistic effect, between −25 and 25 means an additive effect, and a score < 25 indicates an antagonistic effect [64,65]. 

### 3.8. Statistical Analysis

Statistical analysis and graph representation were performed with GraphPad Prism version 8 for Windows (GraphPad Software; San Diego, CA, USA). All data were expressed as mean ± standard deviation (SD) from at least three independent experiments. A Shapiro–Wilk test was conducted to determine the distribution of the samples. To assess the differences between control and treatments, a two-way ANOVA test was used. Statistically significant results are referred to as: * *p* ≤ 0.05, ** *p* ≤ 0.01, *** *p* ≤ 0.001, and **** *p* ≤ 0.0001. 

## 4. Conclusions

This study reported the successful green synthesis of stabilized gold nanoparticles using a *Codium tomentosum* marine seaweed extract to act as a reducing and capping agent. The synthesis resulted in the obtention of monodispersed spherical nanoparticles with a mean size of 34.5 ± 5.6 nm. FTIR analysis confirmed the involvement of hydroxyl and carbonyl groups in the reduction and stabilizing processes, and the analysis of carbohydrates before and after the synthesis suggested that longer chains might be preferentially involved. 

Au@CT significantly decreased cell proliferation in the HEPG-2 cell line, alone and in combination with gemcitabine, inducing a synergistic antitumoral effect. The antiproliferative effect of Au@CT in the BxPC-3 cell line was low and no synergy was detected in combination with gemcitabine, possibly due to the high cytotoxic effect of gemcitabine alone in this cell line.

Our results showed that gold nanoparticles synthesized from seaweed extracts with antitumoral activity could be a good gemcitabine enhancer for the treatment of some tumors. The combination reduced the dose of gemcitabine needed to observe a therapeutic effect in a hepatic cell line. Hence, Au@CT have the potential to decrease the gemcitabine-associated toxicity in the treatment of hepatic cancer.

## Figures and Tables

**Table 1:** mg of carbohydrates belonging to each of the fractions analyzed, occurring in a g of extract.

Fraction	mg/g
>150 kDa	304.4 ± 1
150–50 kDa	80.0 ± 3
50–12 kDa	125.7 ± 1
<12 kDa	208.8 ± 3

## Data Availability

The data used to support the findings of this study are included within the article.

## Supplementary Materials

The following are available online at https://www.mdpi.com/article/10.3390/md21010020/s1, Figure S1: (A) Zeta potential distribution of Au@CT. (B) UV-Vis spectra of Au@CT showing the stability of the samples after 3 months. Figure S2: Kinetics of the cell viability of BxPC-3 incubated with (B) Au@CT (0.4, 4, 40 µM) and (A) CT extract (1, 10, 100 mg/mL) in combination with gemcitabine (0.01, 0.5 µM). The treatments were added after cell stabilization (vertical red lines).
