# Supplementary material for: Gold Nanoparticles Synthesized by an Aqueous Extract of Codium tomentosum as Potential Antitumoral Enhancers of Gemcitabine
Source: Mar Drugs. 2022 Dec 27;21(1):20. doi: 10.3390/md21010020 (PMC9865996; doi:10.3390/md21010020)
Supplement: Supplementary file 1 [file marinedrugs-21-00020-s001.zip › marinedrugs-2101108-supplementary.pdf]

SUPPLEMENTARY INFORMATION

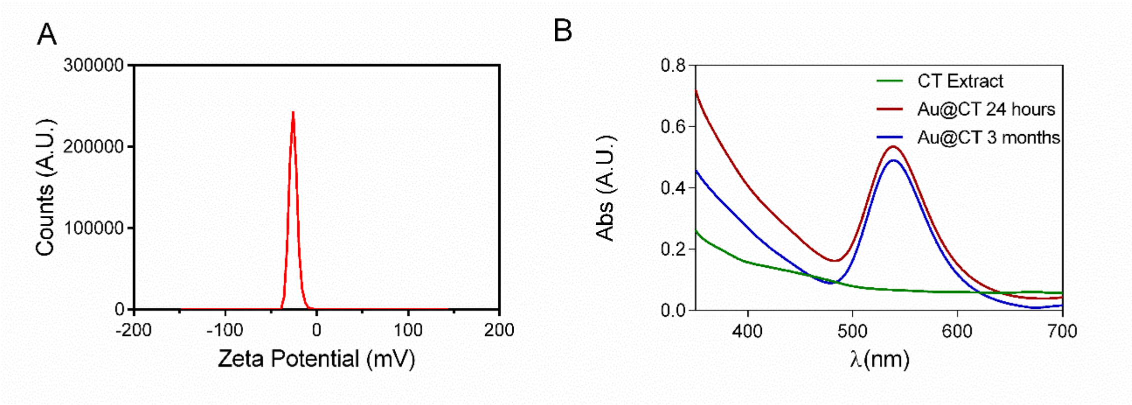

Figure S1. (A) Zeta potential distribution of Au@CT. (B) UV-Vis spectra of Au@CT showing the stability of the samples after 3 months.

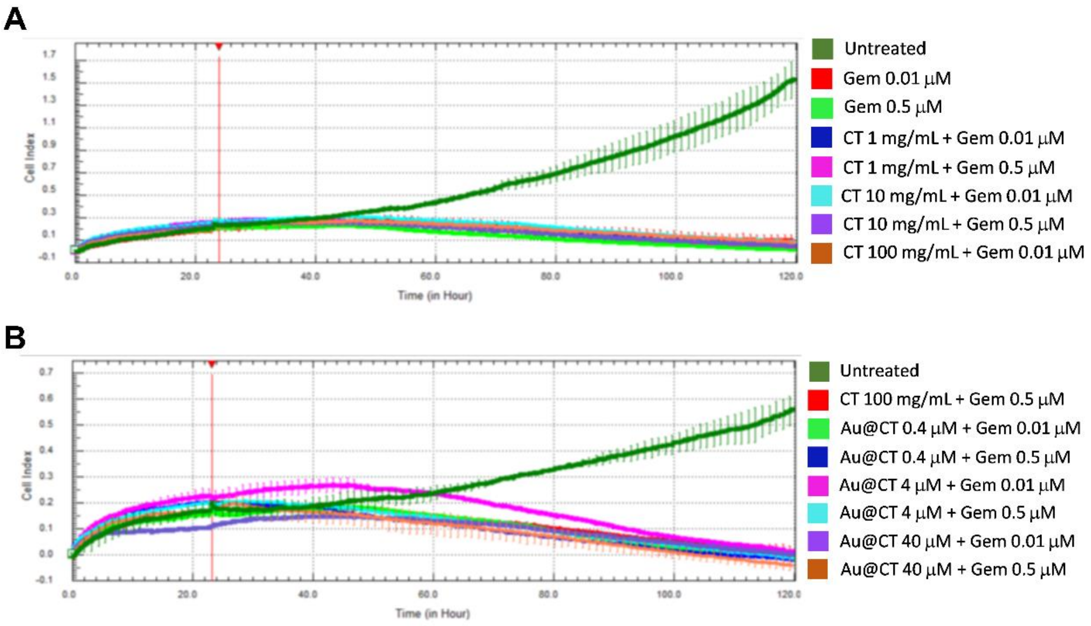

Figure S2. Kinetics of the cell viability of BxPC-3 incubated with (B) Au@CT (0.4, 4, 40 µM) and (A) CT extract (1, 10, 100 mg/mL) in combination with gemcitabine (0.01, 0.5 µM). The treatments were added after cell stabilization (vertical red lines).
